# Hypoxia Inducible Factor 1 Alpha Is Expressed in Germ Cells throughout the Murine Life Cycle

**DOI:** 10.1371/journal.pone.0154309

**Published:** 2016-05-05

**Authors:** Natsumi Takahashi, Philip M. C. Davy, Lauren H. Gardner, Juanita Mathews, Yuki Yamazaki, Richard C. Allsopp

**Affiliations:** Institute for Biogenesis Research, John A Burns School of Medicine, Honolulu, Hawaii, United States of America; Massachusetts General Hospital, UNITED STATES

## Abstract

Pluripotent stem cells of the early embryo, and germ line cells, are essential to ensure uncompromised development to adulthood as well as species propagation, respectively. Recently, the transcription factor hypoxia inducible factor 1 alpha (Hif1α) has been shown to have important roles in embryonic stem cells; in particular, regulation of conversion to glycolytic metabolism and, as we have shown, maintenance of functional levels of telomerase. In the present study, we sought to assess whether Hif1α was also expressed in the primitive cells of the murine embryo. We observed expression of Hif1α in pre-implantation embryos, specifically the 2-cell stage, morula, and blastocyst. Robust Hif1α expression was also observed in male and female primordial germ cells. We subsequently assessed whether Hif1α was expressed in adult male and female germ cells. In the testis, Hif1α was robustly expressed in spermatogonial cells, in both juvenile (6-week old) and adult (3-month old) males. In the ovaries, Hif1α was expressed in mature oocytes from adult females, as assessed both in situ and in individual oocytes flushed from super-ovulated females. Analysis of Hif1α transcript levels indicates a mechanism of regulation during early development that involves stockpiling of Hif1α protein in mature oocytes, presumably to provide protection from hypoxic stress until the gene is re-activated at the blastocyst stage. Together, these observations show that Hif1α is expressed throughout the life-cycle, including both the male and female germ line, and point to an important role for Hif1α in early progenitor cells.

## Introduction

Hallmark features of the primitive progenitor cells of the early embryo include both pluripotency and an extensive capacity to proliferate. The former is attributed to the expression of pluripotency factors, including transcription factors Oct4, Klf4, Sox2 and Nanog [1]. The latter is attributed to maintenance of relatively long telomeres by the enzymatic complex telomerase [2]. However, much remains to be discovered to allow full elucidation of the cell and molecular mechanisms that regulate the function of these cells.

The primitive progenitor cells of the developing embryo include both cells of the pre-implantation embryo, and the inner cell mass of the blastocyst; as well as the early germline stem cells of the embryo, known as primordial germ cells (PGCs), which give rise to both the male and female germ lineages. In murine embryos, PGCs are equivalent for both male and female embryos from 7days post coitus (dpc) through 11dpc [3]. Beginning at 9dpc, PGCs begin to migrate to the developing genital ridge of the embryo, and undergo continuous proliferation to expand the PGC pool. By 13dpc of development the PGCs reside entirely in the developing gonads, and have both committed to sex-specific differentiation and entered a state of quiescence [4]. Shortly after birth, the male germ line resumes proliferation as the testis develop, and the female germ line produces immature oocytes as the ovaries develop. Interestingly, both PGCs and spermatogonial stem cells [5,6] express the pluripotent factor Oct4.

A number of studies have shown that hypoxia promotes pluripotency in both embryonic stem cells (ESC) and induced pluripotent stem cells (iPSC). It has been shown that human ESC (hESC) cultured in hypoxic condition (3–5% O2) exhibit reduced amount of spontaneous differentiation compared to control cells cultured in normoxic condition (21% O2) [7]. When co-cultured with feeder cells overexpressing hypoxia inducible factor 1 alpha (Hif1α), hESC remain undifferentiated and show higher Oct4 and Nanog expressions [8]. It has also been reported that the efficiency of iPSC generation from mouse and human somatic cells is improved in hypoxic environment [9]. More recently, one study has shown that hESC and iPSC derived differentiated cells can return back to a pluripotent state when cultured under hypoxia (2% O2) [10]. Both neural crest stem cells and neural stem cells derived from rats also exhibit increased proliferation and survival in lower oxygen tension [11,12].

Hypoxia occurs when a supply of oxygen decreases and compromises the biological functions. Cells respond to hypoxia by activating one of the key regulators of metabolism, Hif1α. Under normoxic condition, prolyl hydroxylases (PHD) are responsible for hydroxylating a specific proline residue within the oxygen dependent degradation domain of Hif1α. This reaction recruits VHL-ubiquitin-ligase complex to bind to the same region of the Hif1α protein and allows the proteasomal degradation of the protein. However, in the low oxygen environment, the interaction between PHD and Hif1α is inhibited and hydroxylation of Hif1α does not occur. This causes Hif1α to be stabilized and allows the translocation of Hif1α into the nucleus. Once Hif1α is in the nucleus, it dimerizes with HIF1b (ARNT) and binds to specific sites (Hypoxia response element; HRE) allowing regulation of transcription of target genes, such as EPO and VEGF [13].

Hif1α can also be regulated by oxygen-independent means. The RACK1 protein has been shown to mediate Hif1α destruction by binding to Hif1α and recruiting the ubiquitin-ligase complex to Hif1α without the involvement of the VHL [14]. The presence of SSAT1 is known to stabilize the interaction between RACK1 and Hif1α [15]. However, when RACK1 is dephosphorylated by calcineurin A, Hif1α cannot be ubiquitinated due to the failure of RACK1 dimerization, leading to the stabilization of Hif1α [16]. In addition, GSK3 has been shown to down-regulate Hif1α by phosphorylating ODD domain of Hif1α and promoting ubiquitination and proteasomal degradation [17].

The microenvironment, or niche, that stem cells reside in is both specific for different types of stem cells, and critical to the long term regulation of the stem cell pool. Studies have now demonstrated that the niche of some stem cells is hypoxic. For example, hematopoietic stem cells (HSCs) reside in hypoxic regions of the bone marrow [18] and express Hif1α [19,20]. It is also widely accepted that early embryo development occurs in low oxygen environment. Uterine oxygen concentration is known to be significantly low in various mammalian species [21]. Hif1α and HIF2a have also been shown to be involved in trophoblast and placental development [22].

Recently, Hif1α has been shown to play important regulatory roles in stem cells. In embryonic stem cells, Hif1α has been shown to be essential in the conversion of metabolism from aerobic to glycolytic metabolism [23], and also for maintenance of telomerase expression, and stable telomere length [24]. Hypoxia inducible factor 1 also promotes telomere length extension during establishment of iPSC [25]. Furthermore, conditional ablation of Hif1α in murine HSC markedly abrogates long term self-renewal capacity upon serial transplantation [26]. Together, these observations point to an important role for Hif1α in stem cell biology for both pluripotent and adult stem cells.

In this study, we examined whether Hif1α is expressed in primitive stem cells and germ cells in mice. Our results show persistent expression of Hif1α in in the early embryo, PGCs, and in both male and female adult germ cells, suggesting that Hif1α may be involved in the maintenance of germ stem cells.

## Materials and Methods

### Mice

Mice were fed with a standard diet and maintained in a temperature- and light-controlled room (22°C, 14L:10D; light starting at 0700 h), in accordance with the guidelines of the Laboratory Animal Services at the University of Hawaii and the Committee on Care and Use of Laboratory Animals of the Institute of Laboratory Resources National Research Council (DHEW publication 80–23, revised in 1985). The protocol (#03–046) for animal handling and treatment procedures was reviewed and approved by the Animal Care and Use Committee at the University of Hawaii, and conducted in accordance with the Society for the Study of Reproduction’s guidelines and standards.

### FACS isolation of PGCs

Lower halves of 9.5dpc and the gonads of 15.5dpc Oct4-GFP embryos were dissected out and dissociated in 0.05% Trypsin-EDTA (Gibco) for 4 minutes at 37C°. The reaction was stopped with 10% Fetal Bovine Serum (FBS) in Phosphate Buffered Saline (PBS). The cell suspension was filtrated through a 30μm nylon mesh. GFP+ cells were FACS sorted using FACSAria III (BD Biosciences) following identification of the single cell population by forward scatter gating and removal of dead cells by gating cells negative for 7-Aminoactinomycin D staining.

### Primary and secondary antibodies

The primary antibodies used were as follows: anti-HIFα rabbit polyclonal (1:200 for preimplantation embryos and 1:500 for the others, cat# NB100-479, Novus Biologicals); anti-Oct3/4 mouse monoclonal (1:200, cat# sc-5279, Santa Cruz Biotechnology); and anti-GFP mouse monoclonal (1:500, cat# G6539, Sigma-Aldrich). The following secondary antibodies were used at a dilution of 1:1000: DyLight 594 goat anti-rabbit IgG (cat# DI-1594, Vector Laboratories); DyLight 488 horse anti-mouse IgG (cat# DI-2488, Vector Laboratories); FITC-conjugated goat anti-mouse IgG2b (cat# ab98702, Abcam); and biotinylated donkey anti-rabbit IgG (cat# 711-065-152, Jackson ImmunoResearch).

### Whole-mount staining

For whole-mount embryo staining, preimplantation embryos and 9.5dpc embryos were fixed in 4% paraformaldehyde (PFA) at room temperature (RT) for 30 minutes and 2 hours, respectively. Preimplantation embryos were incubated with primary antibodies overnight at 4C° and with secondary antibodies for 2 hrs at RT. 9.5dpc embryos were incubated with primary antibodies for 72 hours at 4C° and with secondary antibodies overnight at 4C°. Both preimplantation embryos and 9.5dpc embryos were mounted in VECTASHIELD (Vector Laboratories) for observation by confocal microscopy (LSM 5 PASCAL, Zeiss). A total 14 pre-implantation embryos and eight 9.5dpc embryos were assessed.

### Immunofluorescence staining

For immunostaining of 15.5dpc gonads, the testes and ovaries were fixed in 4% PFA overnight at 4C°, placed in 30% sucrose, embedded in OCT compound and cryosectioned at 10μm. The cryosections were incubated with primary antibodies for 2 hours at RT, with secondary antibodies for 90 minutes at RT and mounted with in VECTASHIELD. Images were acquired with a fluorescence microscope (BX41, Olympus). A total of eight male and female 15.5dpc gonads and 12 ovaries from female mice were examined, and at least 6 fields of view were analyzed per sample.

### DAB immunoperoxidase staining

For immunoperoxidase staining of gonads, the adult testes were fixed in Bouin’s fixative overnight at 4C°. The tissues were embedded in paraffin and sectioned at 5μm. The sections were incubated with primary antibody for 2 hours at RT and with biotinylated secondary antibody for 30 minutes at RT. R.T.U. Vectastain ABC kit and ImmPACT DAB peroxidase substrate (Vector Laboratories) were used for detection. A total of five testes were examined, and ten fields of view analyzed per sample.

### Immunocytochemistry

FACS sorted GFP+ cells from 9.5dpc and 15.5dpc embryos were placed on the slides by cytospinning and were fixed in 4% PFA for 15 minutes at RT. The slides were incubated with primary antibodies for 2 hours at RT and with secondary antibodies for 90 minutes at RT and mounted in VECTASHIELD with DAPI. A total of ten fields of view were assessed per sample.

### Western blot analysis

Tissues and deferoxamine treated cells were homogenized in lysis buffer (50mM Tris (pH 7.6), 150mM NaCl, 80mM NaF, 1 mM PMSF (phenylmethylsulfonyl fluoride), 1:100 protease inhibitor cocktail (Sigma P8340), and 0.5% Nonidet P-40) and incubated on ice for 20 minutes. Cellular debris was separated by centrifugation at 10,000 x g for 5 minutes at 4°C. The supernatant’s protein content was quantified using a BCA assay (Pierce). 50μg of protein was loaded on a 6% SDS-PAGE gel. Protein was transferred from gel to PVDF membrane then blocked with 5% milk in TBS-T for 2 hours at room temp. Membrane was then put into primary antibody against Hif1α (NB100-134, Novus Biologicals) diluted in blocking solution at 1:1000 for 1 hour. Membrane was then washed 3 times 10 minutes each with TBS-T. Secondary antibody (horseradish peroxidase-conjugated anti-rabbit IgG) was then added for 1 hour at a 1:100,000 dilution in blocking solution. Membrane was then washed an additional 3 times for 10 minutes each with TBS-T. Advanced ECL kit (Amersham) was applied to the membrane for 5 minutes and then membrane was exposed to film.

### Reverse Transcriptase—Polymerase Chain Reaction

Oocytes were collected following hormone-induced ovulation of BA mice; blastocysts were likewise collected 3.5 days after timed-mating of the same. Primordial germ cells were collected at 9.5 dpc from Oct4-GFP mice by FACS sorting as described above. 100 oocytes, 20 blastocysts, and 20,000 PGCs were processed with the CellsDirect One-Step qRT-PCR Kit (LifeTechnologies) directly to cDNA, Total RNA was extracted from 100 ug of testes tissue, and mouse embryo fibroblast (MEF) cells exposed to 2% oxygen for 6 hours, with TRIZOL then converted to cDNA with an iScript cDNA Synthesis Kit (Bio-Rad). NoRT reactions were run concurrently with the processing of each sample. RT-PCR was performed using Hypoxanthine-guanine phosphoribosyltransferase (HPRT) and Hif1α primers designed to span exon-exon junctions in 25ul reactions with 2ul of oocyte, blastocyst, and PGC cDNA and 100ng of testes or MEF cDNA per reaction. The PCR products were resolved on 3% agarose gels in 1X Sodium Borate buffer with ethidium bromide staining.

## Results

### Hif1α expression in preimplantation embryos

**Fig 1:**
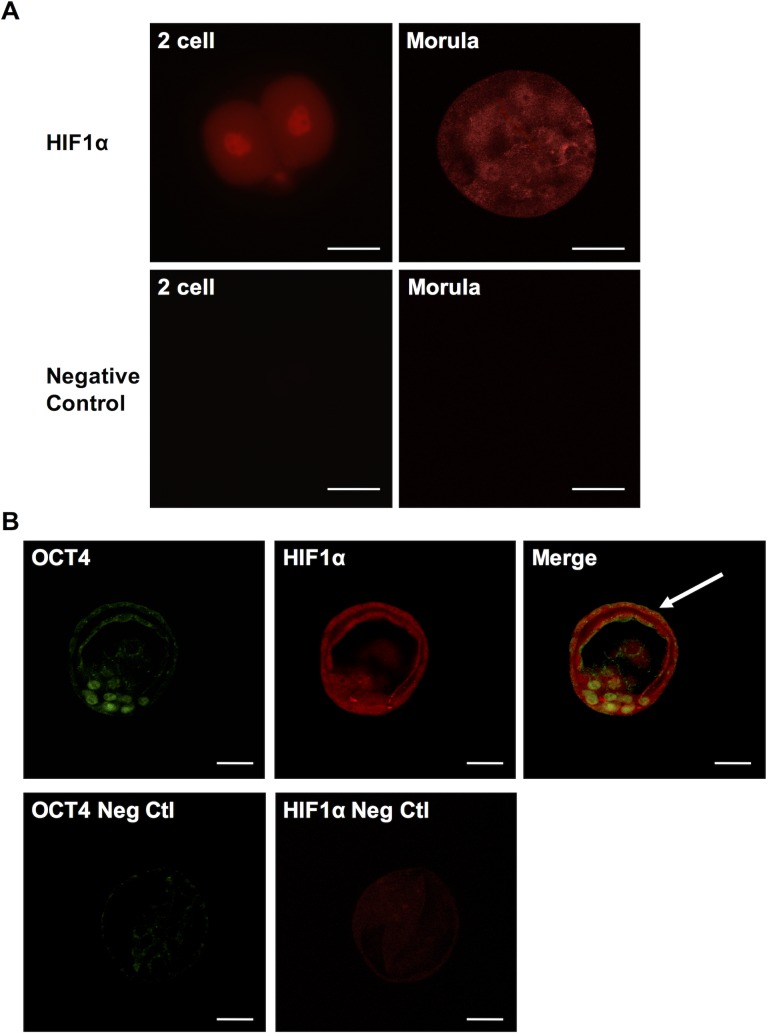
Hif1α expression in preimplantation embryos. (A) Hif1α staining in two-cell stage embryo and morula (top) and negative control images without primary antibody (bottom). (B) Co-staining of OCT4 and Hif1α in inner cell mass of blastocyst (top) and negative control images without primary antibodies (bottom). Scale bar: 30μm.

Hif1α is readily detectable in murine embryonic stem cell cultures [27]. To assess if this reflects true physiological expression of Hif1α, we measured Hif1α levels in preimplantation embryos at three different stages– 2-cell embryo, morula and blastocyst. Following superovulation with PMSG and hCG and timed mating, embryos were harvested by flushing out the oviducts and the uterus. Whole-mount immunostaining of the embryos showed Hif1α expression in both cytoplasm and nucleus of two-cell stage embryo at 1.5dpc as well as in morula at 3.5dpc (Fig 1A). Hif1α was also detected in the OCT4-positive inner cell mass of blastocysts at 4.0dpc (Fig 1B). Interestingly, unlike Oct4 expression, we also detected Hif1α expression in the trophectoderm (Fig 1B).

### Hif1α expression in migratory PGCs

**Fig 2:**
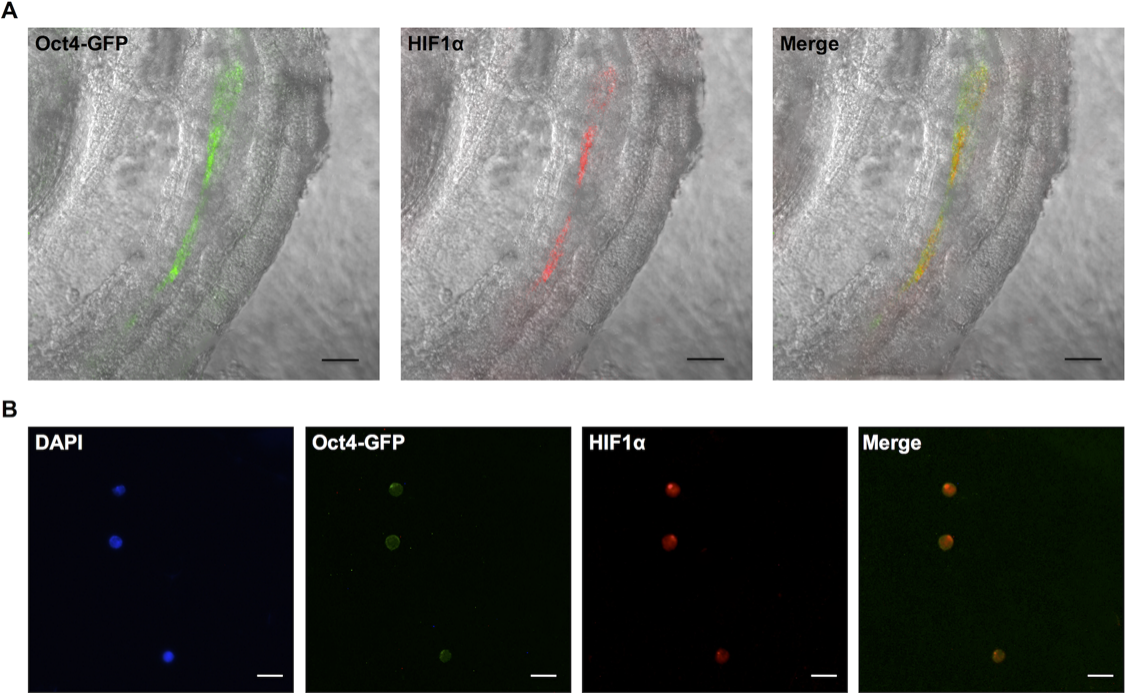
Hif1α expression in 9.5dpc primordial germ cells. (A) Whole-mount staining of Hif1α and GFP in Oct4-GFP embryo (lateral view). Scale bar: 100μm. (B) Hif1α and GFP staining of FACS-sorted PGCs. Scale bar: 20μm.

We then examined whether Hif1α persists in the primordial stem cell compartment in more developed embryo at 9.5dpc. Here we utilized the Oct4-GFP transgenic strain [28], in which GFP expression is driven by Oct4 promoter. Previous work has shown that nearly all GFP+ cells obtained from the Oct4-GFP transgenic embryos at 9.5dpc are PGCs [28]. At this stage of development, male and female PGCs are equivalent, and are actively migrating from the hindgut towards the developing gonadal ridges (reviewed by Richardson & Lehmann, 2010[29]). We performed whole-mount staining of these embryos and observed co-localization of GFP and Hif1α in migrating PGCs (Fig 2A). FACS sorted GFP+ cells from d9.5 Oct4-GFP embryos also exhibited co-localization of GFP and Hif1α as expected (Fig 2B).

### Hif1α expression in developing gonads

**Fig 3:**
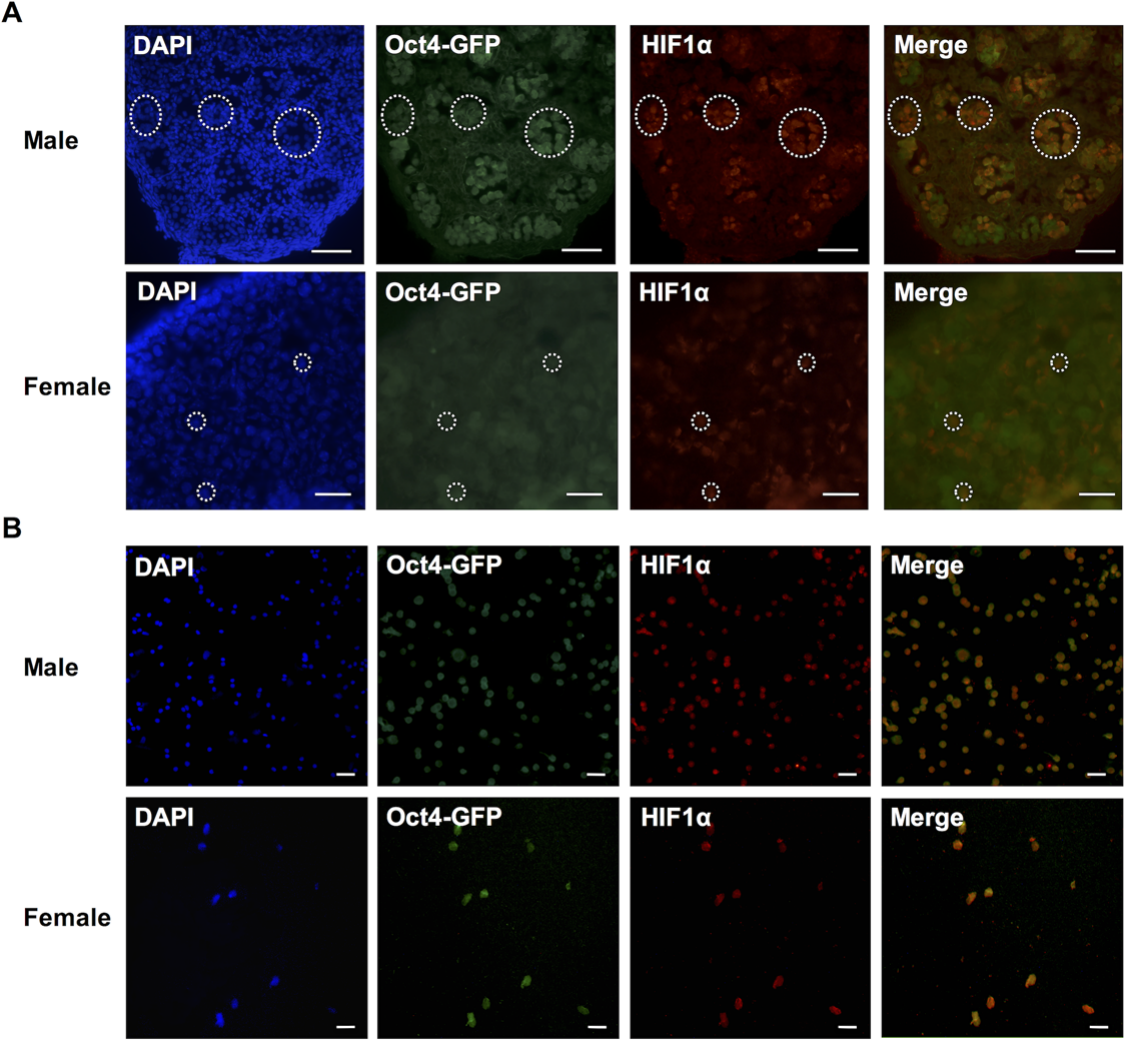
Hif1α expression in 15.5dpc germ cells. (A) Sections of male (top) and female (bottom) gonads from 15.5 dpc Oct4-GFP embryos showing Oct4-GFP and Hif1α expressions in germ cells. Scale bars: 50μm (male) and 20μm (female). (B) FACS-sorted male (top) and female (bottom) 15.5dpc germ cells showing Oct4-GFP and Hif1α expressions. Scale bars: 100μm (male) and 50μm (female).

We investigated whether the expression of Hif1α observed in the primitive germline at 9.5dpc continues to be present after PGCs reach the developing gonads and commit to either the male or female germ lineage. Both male and female gonads from 15.5dpc Oct4-GFP embryos were used for this experiment. Immunostaining of cryo-sections revealed that Hif1α is expressed in male PGCs located within the testis cords at 15.5dpc (Fig 3A top). Hif1α was also detected in female PGCs within germ cell cysts (Fig 3A bottom). We also FACS sorted GFP+ cells from 15.5dpc Oct4-GFP male and female gonads and observed the co-localization of GFP and Hif1α in both (Fig 3B).

### Hif1α expression in the neonatal testis and adult testis

**Fig 4:**
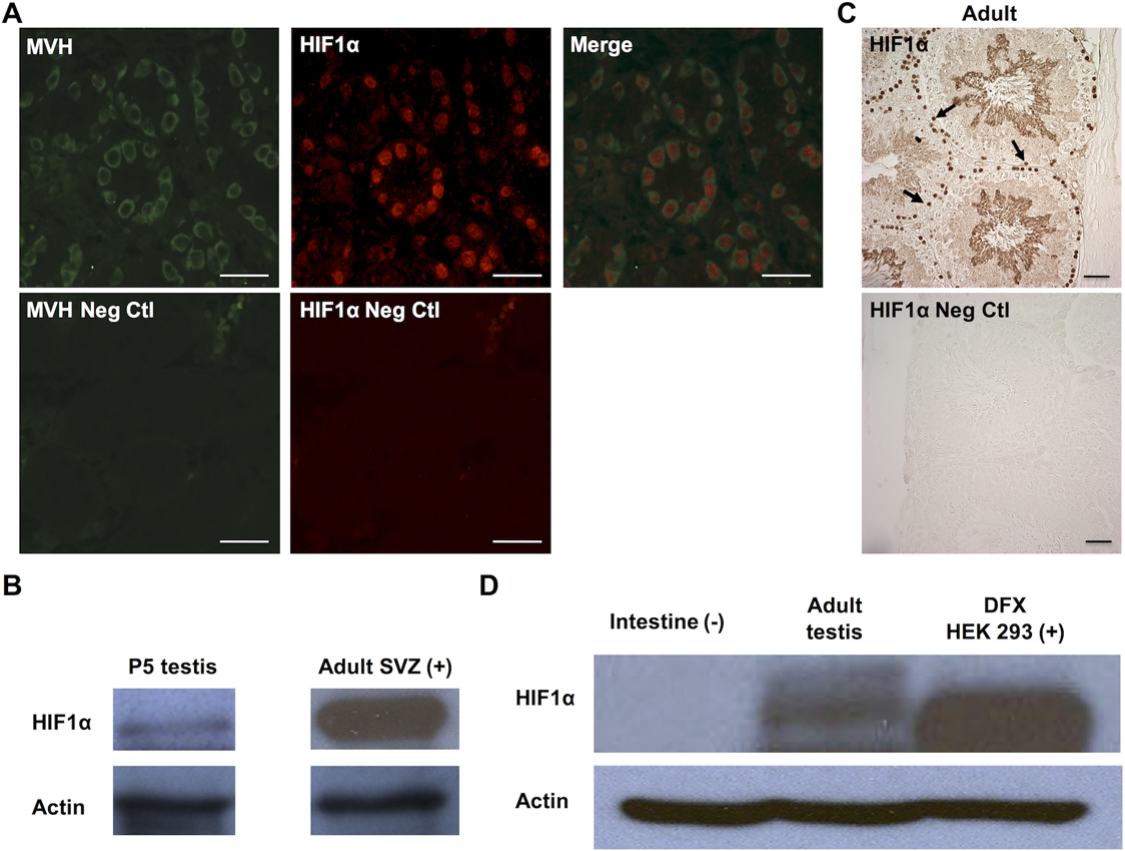
Hif1α expression in neonatal and adult testis. (A) Section of testis from 5-day old (P5) male new born pups showing Hif1α expression in MVH+ gonocytes within the seminiferous tubules (top) and negative control images without primary antibodies (bottom). Scale bar: 50μm. (B) Western blot analysis of Hif1α expression in P5 testes (left) compared to extract of the adult brain sub-ventricular zone (SVZ) (right). Loading control (β-Actin) is shown below. (C) Section of adult (3 month old) testis showing Hif1α expression in spermatogonia. Scale bar: 30μm. (D) Western blot analysis of whole adult testis. HEK293 cells treated with DFX were used as a positive control and intestinal tissue was used as a negative control. Loading control (β-Actin) is shown below.

To assess whether Hif1α expression persists in the developing gonads postpartum, we next examined Hif1α expression pattern in neonatal gonads. Double staining of P5 testis with Hif1α and a cytoplasmic germ cell marker, mouse VASA homolog (MVH), revealed Hif1α expression in gonocytes within the seminiferous tubules (Fig 4A). We also performed western blot analysis of whole P5 testis and confirmed an abundant level of Hif1α expression at this stage (Fig 4B).

To assess whether Hif1α expression continues in adult male germ cells, we performed immunostaining of testis from adult (three-month old) male mice and observed Hif1α expression in the nucleus of spermatogonial population (Fig 4C). The same expression pattern was also observed in testis from young adult mice (six-week old) testis (S1 Fig). We performed western blot analysis of whole adult testis and confirmed the presence of abundant levels of Hif1α in the adult testis; human embryonic kidney 293 (HEK293) cells with Hif1α stabilization achieved by treatment with the iron chelator deferoxamine (DFX), a hypoxia mimetic, were used as a positive control (Fig 4D).

### Hif1α expression in the neonatal and adult ovary

**Fig 5:**
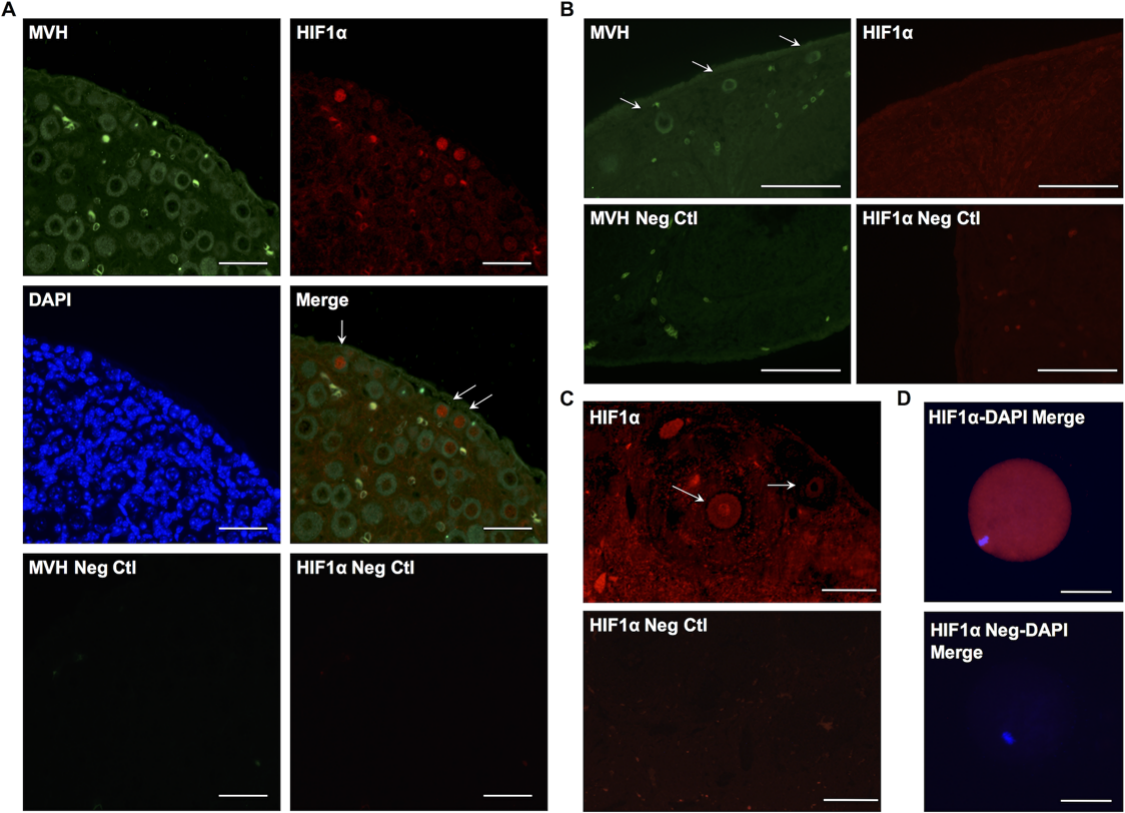
Hif1α expression in neonatal and adult ovary. (A) Section of P5 ovary showing MVH expression in all oocytes and Hif1α expression only in small oocytes (primary follicles; top). Negative control images without primary antibodies (bottom). Scale bar: 50μm. (B) Section of adult ovary showing absence of Hif1α expression in primary follicles detected with MVH staining (top). Negative control without primary antibody (bottom). Scale bar: 50μm. (C) Section of adult ovary showing Hif1α expression in mature primary oocyte (arrow). Negative control without primary antibody (bottom). Scale bar: 100μm. (D) Image of a Metaphase II oocyte from superovulated three-month-old female showing Hif1α expression. Scale bar: 50μm.

Double staining of P5 ovary with Hif1α and MVH revealed Hif1α expression in these early stage oocytes, and at particularly high levels in the nuclei of very small oocytes located in the cortical region of the neonatal ovary (Fig 5A). However, Hif1α was not detected in larger oocytes found towards the center of the tissue.

In contrast, we did not detect Hif1α localization to smaller oocytes in the adult ovary (Fig 5B), rather, Hif1α expression was observed in larger, mature oocytes from ovaries of mice 6 weeks and 3 months of age (S1 Fig and Fig 5C). These Hif1α positive oocytes from adult mice are surrounded by several layers of granulosa cells, an indication of a more mature state of development than the smaller Hif1α-positive oocytes found in the neonatal ovary. To determine whether Hif1α expression continues in the oocyte after ovulation (Metaphase II oocyte), we performed immunostaining of oocytes collected from adult (3 month-old) female mice that had been superovulated with PMSG and hCG. We observed a very clear, robust expression of Hif1α throughout the metaphase II oocyte (Fig 5D).

### Regulation of Hif1α expression during early development

**Fig 6:**
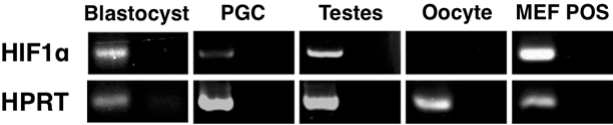
Transcription of Hif1α during early stages of development. RT-PCR analysis of Hif1α transcription in 3.5 dpc blastocysts, 9.5 dpc PGCs, whole testes tissue, mature oocytes, and hypoxic-cultured MEFs. Hif1α PCR products are shown in the upper row with HPRT single-copy gene control reactions in the bottom row, cDNA reactions are shown with complementary noRT reactions for each gene and sample (left and right respectively).

To begin to assess the regulatory mechanisms controlling Hif1α expression during early development, we performed RT-PCR analysis of Hif1α mRNA in blastocysts, 9.5 dpc PGCs, whole testes, and mature oocytes. Mouse embryonic fibroblast (MEF) cells exposed to hypoxic conditions were used as a positive control for Hif1α expression. Blastocysts, PGCs, and testes all showed the presence of Hif1α transcript, while mature oocytes did not (Fig 6), despite the abundant presence of the protein (Fig 5D). This suggests that the Hif1α protein is stockpiled in mature oocytes, to provide sufficient reserves of Hif1α in the hypoxic microenvironment of the early embryo, until the gene is re-activated, at approximately the blastocyst stage of development.

## Discussion

Hif1α is a key regulator for a number of adaptive responses to hypoxia, such as changes in energy metabolism and stimulation of angiogenesis. In addition, Hif1α has also been shown to be involved in maintenance of various types of stem and progenitor cells, including ESCs [30], neural stem cells [31], mesenchymal stem cells [32] and HSCs [20]. However, the expression and regulation of Hif1α in primitive stem cells and the germ cell compartment has not been extensively studied. Interestingly, our results show that Hif1α is expressed throughout the life cycle in mice, throughout early development, in PGCs, and in adult germ cells, suggesting that hypoxia and Hif1α may play an important role in early development, the maturation of germ cells, as well as the maintenance of germ stem cells.

Immunohistochemical analyses revealed that Hif1α is expressed in pre-implantation embryos at two cell, morula and blastocyst stages (Fig 1A and 1B). In blastocysts, the OCT4-positive, pluripotent inner cell mass expresses Hif1α. This observation agrees with the findings from our previous study showing that Hif1α is present in mouse ESC cultured under normoxic condition [24]. Hif1α continues to be expressed in migrating PGCs at 9.5 dpc before they are committed to a germ cell lineage (Fig 2), and persists in both male and female PGCs at 15.5 dpc (Fig 3) as well as in neonatal reproductive tissues (Figs 4 and 5).

In adult testis, the expression of Hif1α is detected in the spermatogonial stem cell compartment along the basement membrane of the seminiferous tubules. As those cells differentiate into spermatocytes, Hif1α signal becomes less visible by immunohistochemical assay. Towards the end of spermatogenesis, specifically during the process of spermiogenesis, Hif1α is again detected in the elongated spermatids (Fig 4C). As it has been shown that the activity of telomerase is the highest in the spermatogonial stem cells and decreases as the cells differentiate [33], and also that Hif1α regulates telomerase activity and telomere length in mouse ESC [24], it is possible that Hif1α is involved in the maintenance of spermatogonial stem cells by up-regulating telomerase, or more specifically, telomerase reverse transcriptase. Our observation of Hif1α expression in elongated spermatids, as well as the previously reported Hif1α expression in the mature spermatozoa [34] suggest that Hif1α might also play a protective role in haploid male germ cells. Further evaluation is necessary in order to assess the mechanisms for Hif1α regulation as well as possible Hif1α function in spermatogenic stem cells and during spermatogenesis.

In neonatal ovary, we detected Hif1α in small oocytes in the cortical region, but not in larger oocytes found towards the center of the ovary (Fig 5). However, in adult ovaries, Hif1α is detected in more mature oocytes located within secondary and antral follicles and not in smaller oocytes (Fig 5). It is possible that small Hif1α-positive oocytes in the neonatal ovary still reside within the germ cell cysts, which would explain why we did not observe any Hif1α-positive small oocytes in adult ovary because the breakdown of the germ cell cysts into individual follicles, completed several days after birth, would cause the leakage of Hif1α protein out of the cells.

There are a number of intriguing roles Hif1α may play in primitive stem cells and germ cells. Given the importance of telomerase in maintenance of telomeres in the early embryo, and in germ cells, particularly male germ cells, Hif1α may play a role in regulation of telomerase, as we have observed previously in murine ESC [24]. Indeed, telomerase activity is readily detected in male gonads [35], PGCs [36], and primitive cells in the early embryo [37]. The role of Hif1α in these cells certainly goes beyond regulation of telomerase. In ESC and iPSC Hif1α is required to switch energy metabolism from oxygen-dependent respiration to glycolysis, which may have fundamental roles in the maintenance of pluripotency [23]. Furthermore, other studies have linked Hif1α to the oxidative stress response pathway involving FOXO3 [38,39].

Hif1α levels may also be regulated at translational level. It has been shown that IGF-1 increases Hif1α synthesis through PI3K and MAPK pathways in colon cancer cells [40]. In kidney cancer cells, the ability of mTOR to promote Hif1α translation has also been studied [41]. Therefore, it is possible that the expression of Hif1α we observed throughout the murine lifecycle is regulated at translational level, although the mechanism might be different at various developmental stages and also between sexes. In support of this notion, we have observed stable and abundant Hif1α protein in mature oocytes in the absence of detectable Hif1α transcript (Fig 6). This likely reflects an oxygen independent stabilization of Hif1α. This high level of Hif1α in oocytes presumably serves as a reserve to last until the *Hif1α* gene is re-activated at around the blastocyst stage.

In summary, the present study shows that Hif1α is expressed throughout the life cycle of mouse germ cells, indicating the importance of Hif1α during normal development of germ cells. It is likely that Hif1α has multiple functions during the process of germ cell development, considering the fact that it is present in both male and female germ cells. Future experiments using a germ cell specific Hif1α knockout mouse model will allow us to evaluate the specific roles of Hif1α in germ cell development.

## Supporting Information

S1 Fig Analysis of Hif1α expression in young mice. Sections of 6-week-old testis and ovary showing Hif1α expression in spermatogonia (left) and oocytes (right). Scale bar: 50μm.
(TIF)
