# Supplementary figures and images for: Hypoxia Inducible Factor 1 Alpha Is Expressed in Germ Cells throughout the Murine Life Cycle
Source: PLoS One. 2016 May 5;11(5):e0154309. doi: 10.1371/journal.pone.0154309 (PMC4858237; doi:10.1371/journal.pone.0154309)

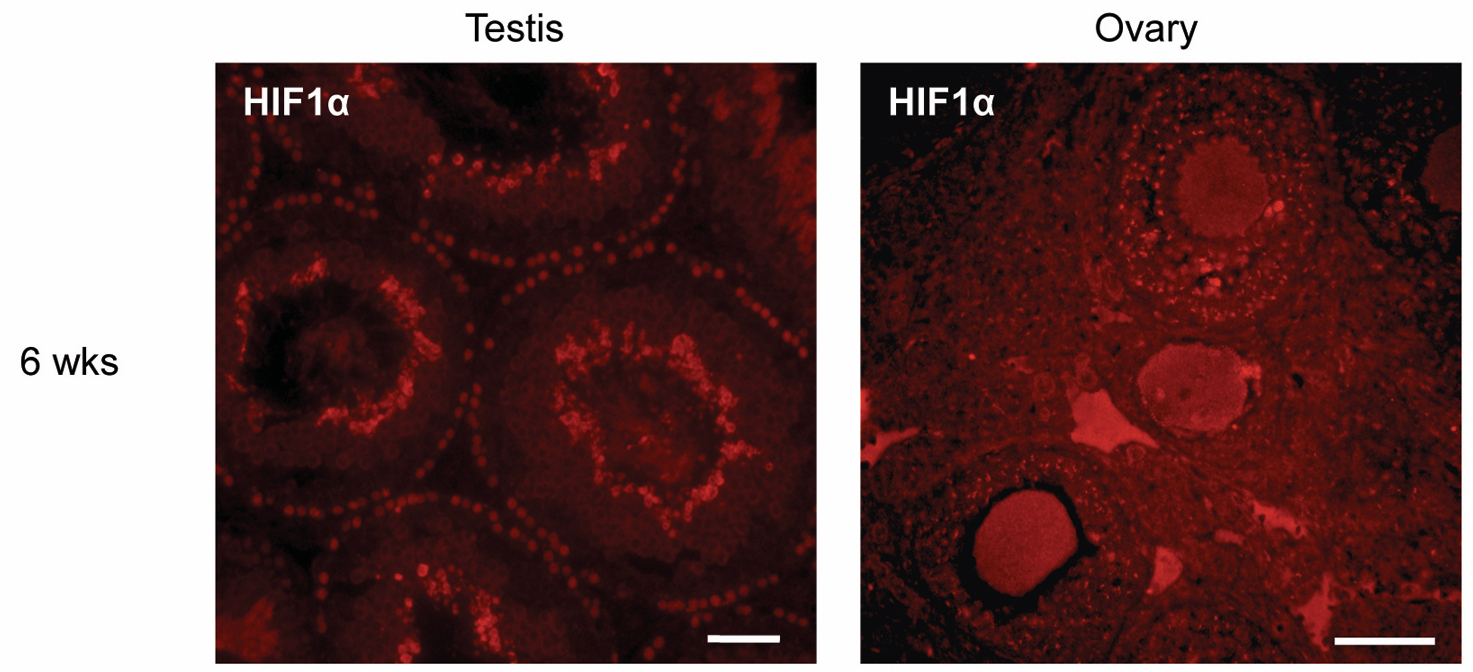

Supplement: S1 Fig — Sections of 6-week-old testis and ovary showing Hif1α expression in spermatogonia (left) and oocytes (right). Scale bar: 50μm. (TIF) [file pone.0154309.s001.tif]
